# Creating a plasma coordination center to support COVID-19 outpatient trials across a national network of hospital blood banks

**DOI:** 10.1017/cts.2024.642

**Published:** 2024-11-14

**Authors:** Anusha Yarava, Christi Marshall, David E. Reichert, Aaron Ye, Preeti Khanal, Sanford H. Robbins, Bruce S. Sachais, David Oh, Ryan A. Metcalf, Kathleen Conry-Cantilena, Karen King, Meredith Reyes, Jill Adamski, Marisa B. Marques, Minh-Ha Tran, Elizabeth S. Allen, Daniel Pach, Neil Blumberg, Rhonda Hobbs, Tammon Nash, Aarthi G. Shenoy, Giselle S. Mosnaim, Yuriko Fukuta, Bela Patel, Sonya L. Heath, Adam C. Levine, Barry R. Meisenberg, Shweta Anjan, Moises A. Huaman, Janis E. Blair, Judith S. Currier, James H. Paxton, William Rausch, Kevin Oei, Matthew Abinante, Donald N. Forthal, Martin S. Zand, Seble G. Kassaye, Edward R. Cachay, Kelly A. Gebo, Shmuel Shoham, Arturo Casadevall, Nichol A. McBee, Daniel Amirault, Ying Wang, Erica Hopkins, David M. Shade, Oliver Layendecker, Sabra L. Klein, Han-Sol Park, John S. Lee, Patrizio Caturegli, Jay S. Raval, Daniel Cruser, Alyssa F. Ziman, Jonathan Gerber, Thomas J. Gniadek, Evan M. Bloch, Aaron A.R. Tobian, Daniel F. Hanley, David J. Sullivan, Karen Lane, the CSSC (COVID 19 Serologic Studies Consortium) group

**Affiliations:** 1 Department of Neurology, BIOS Clinical Trial Coordinating Center, Trial Innovation Center, Johns Hopkins University School of Medicine, Baltimore, MD, USA; 2 Department of Pathology, Johns Hopkins University School of Medicine, Baltimore, MD, USA; 3 Department of Pathology, Luminis Health, Anne Arundel Medical Center Blood Center, Annapolis, MD, USA; 4 New York Blood Center Enterprises, New York, NY, USA; 5 Hoxworth Blood Center, University of Cincinnati, Cincinnati, OH, USA; 6 Department of Pathology, University of Utah School of Medicine, Salt Lake City, UT, USA; 7 Department of Pathology, MedStar Washington Hospital Center, Washington, DC, USA; 8 Department of Pathology, Rhode Island Hospital, Brown University, Providence, RI, USA; 9 Department of Pathology, Baylor College of Medicine, Houston, TX, USA; 10 Department of Laboratory Medicine and Pathology, Mayo Clinic Hospital, Phoenix, AZ, USA; 11 Department of Pathology, University of Alabama at Birmingham, Birmingham, AL, USA; 12 Department of Pathology, University of California, Irvine, CA, USA; 13 Department of Pathology, University of California, San Diego, CA, USA; 14 Department of Pathology, Hoag Memorial Hospital, Newport Beach, CA, USA; 15 Department of Department of Pathology and Laboratory Medicine, Transfusion Medicine Division, University of Rochester Medical Center, Rochester, NY, USA; 16 Department of Laboratory Medicine, Memorial Hermann Texas Medical Center, Houston, TX, USA; 17 Department of Pathology, Wayne State University, Detroit, MI, USA; 18 Department of Medicine, Division of Hematology and Oncology, MedStar Washington Hospital Center, Washington, DC, USA; 19 Division of Allergy and Immunology, Department of Medicine, Endeavor Health, Evanston, IL, USA; 20 Department of Medicine, Section of Infectious Diseases, Baylor College of Medicine, Houston, TX, USA; 21 Department of Medicine, Division of Pulmonary and Critical Care Medicine, University of Texas Health Science Center, Houston, TX, USA; 22 Department of Medicine, Division of Infectious Diseases, University of Alabama at Birmingham, Birmingham, AL, USA; 23 Department of Emergency Medicine, Rhode Island Hospital, Brown University, Providence, RI, USA; 24 Department of Medicine, Luminis Health, Annapolis, MD, USA; 25 Department of Medicine, Division of Infectious Diseases, University of Miami Miller School of Medicine, Miami, FL, USA; 26 Department of Medicine, Division of Infectious Diseases, University of Cincinnati, Cincinnati, OH, USA; 27 Department of Medicine, Division of Infectious Diseases, Mayo Clinic Hospital, Phoenix, AZ, USA; 28 Department of Medicine, Division of Infectious Diseases, University of California, Los Angeles, CA, USA; 29 Department of Emergency Medicine, Wayne State University School of Medicine, Detroit, MI, USA; 30 Nuvance Health, Danbury, CT, USA; 31 Ascada Research, Fullerton, CA, USA; 32 Department of Medicine, Division of Infectious Diseases, University of California, Irvine School of Medicine, Irvine, CA, USA; 33 Department of Medicine, University of Rochester Medical Center, Rochester, NY, USA; 34 Department of Medicine, Division of Infectious Diseases, Georgetown University Medical Center, Washington, DC, USA; 35 Department of Medicine, Division of Infectious Diseases, University of California, San Diego, CA, USA; 36 Department of Medicine, Division of Infectious Diseases, Johns Hopkins University School of Medicine, Baltimore, MD, USA; 37 W. Harry Feinstone Department of Molecular Microbiology and Immunology, Johns Hopkins Bloomberg School of Public Health, Baltimore, MD, USA; 38 Yale Center for Clinical Investigation, Yale University School of Medicine, New Haven, CT, USA; 39 Aerotek, Hanover, MD, USA; 40 Department of Epidemiology, Johns Hopkins Bloomberg School of Public Health, Baltimore, MD, USA; 41 Division of Intramural Research, National Institute of Allergy and Infectious Diseases, Bethesda, MD, USA; 42 Department of Pathology, University of New Mexico School of Medicine, Albuquerque, NM, USA; 43 Nuvance Health Vassar Brothers Medical Center, Poughkeepsie, NY, USA; 44 Department of Pathology and Laboratory Medicine, David Geffen School of Medicine, University of California, Los Angeles, CA, USA; 45 Department of Medicine, Division of Hematology and Oncology, University of Massachusetts Chan Medical School, Worcester, MA, USA and Perlmutter Cancer Center, NYU Langone Health, New York, NY, USA; 46 Department of Pathology and Laboratory Medicine, Endeavor Health, Evanston, IL, USA

**Keywords:** COVID-19, convalescent plasma, decentralized clinical trial, clinical trial management, supply chain management, investigational drug services, cloud-based platform

## Abstract

**Introduction:**

In response to the COVID-19 pandemic, we rapidly implemented a plasma coordination center, within two months, to support transfusion for two outpatient randomized controlled trials. The center design was based on an investigational drug services model and a Food and Drug Administration-compliant database to manage blood product inventory and trial safety.

**Methods:**

A core investigational team adapted a cloud-based platform to randomize patient assignments and track inventory distribution of control plasma and high-titer COVID-19 convalescent plasma of different blood groups from 29 donor collection centers directly to blood banks serving 26 transfusion sites.

**Results:**

We performed 1,351 transfusions in 16 months. The transparency of the digital inventory at each site was critical to facilitate qualification, randomization, and overnight shipments of blood group-compatible plasma for transfusions into trial participants. While inventory challenges were heightened with COVID-19 convalescent plasma, the cloud-based system, and the flexible approach of the plasma coordination center staff across the blood bank network enabled decentralized procurement and distribution of investigational products to maintain inventory thresholds and overcome local supply chain restraints at the sites.

**Conclusion:**

The rapid creation of a plasma coordination center for outpatient transfusions is infrequent in the academic setting. Distributing more than 3,100 plasma units to blood banks charged with managing investigational inventory across the U.S. in a decentralized manner posed operational and regulatory challenges while providing opportunities for the plasma coordination center to contribute to research of global importance. This program can serve as a template in subsequent public health emergencies.

## Introduction

Blood banks and donor centers have remarkable safety records, work under stringent regulatory oversight, and keep blood safe with screening tests for the major transfusion-transmitted infections [1]. Blood banks are organized for accurate and detailed record keeping by tracking every blood product from the point of collection to patient transfusion [2]. Blood banks in academic medical centers may occasionally manage blood products for research trials; however, most lack experience in executing clinical trials that require careful blinding and randomization of interventional blood products. The COVID-19 pandemic and investigational use of COVID-19 convalescent plasma (CCP) for treatment required rapid implementation of new protocols to support CCP clinical trials. Although convalescent plasma has been used to treat infectious diseases for more than 100 years, few contemporary studies were available to model a national qualification, acquisition, and distribution coordination center.

When blood products are used for research trials, various distribution models have emerged for handling trial logistical needs. One example was an open-label study of influenza convalescent plasma, directed by the NIH, between 2011 and 2015. Hospitalized participants (*n* = 98) were randomized to receive either investigational product (IP) or standard of care (no control, saline or plasma product) [3]. Initially, the convalescent plasma in that study was collected from blood donors and qualified through pre-collection antibody screening protocols; then, the strategy transitioned to screening all donor units for high viral-specific antibodies at the Frederick National Laboratory for Cancer Research that also acted as the central NIH donor unit repository for storage and shipping to sites [4].

Most of the CCP trials have been restricted to hospital inpatient transfusions; only a few studies have included outpatient transfusions. Under the Food and Drug Administration (FDA)’s COVID-19 expanded access protocol, CCP was collected by blood establishments and hospital-based donor centers for more than 100,000 open-label inpatient transfusions but it did not have the infrastructure to support complex clinical trials [5]. The University of Pennsylvania collected CCP units and transfused 80 hospitalized participants at a single hospital. The Veterans Administration randomized 75 hospitalized participants to receive CCP or saline control using the large blood services provider Vitalant (Scottsdale, AZ) to handle central storage and distribution to 23 study sites [6,7]. The NHLBI-sponsored Clinical trial of COVID-19 Convalescent Plasma in Outpatients (C3PO) also utilized Vitalant to qualify and distribute CCP to 48 emergency departments in 21 states, transfusing 511 participants [8]. In an outpatient CCP study in Argentina, two unblinded infusion teams drove from a central blood service where the CCP was stored to transfuse 160 participants at 15 Buenos Aires hospitals [9].

Our coordination center design was based on an investigational drug services (IDS) model [10]. IDS pharmacies are specialized units in individual clinical research settings that manage IP for clinical trials. They ensure regulatory compliance and establish standardized procedures for the procurement, storage, handling, and dispensing of these products. Additionally, IDS pharmacies implement safety and control measures to maintain product quality and keep meticulous records to ensure traceability and accountability throughout the trial process.

We then extended the IDS model into an investigational drug (plasma) coordination center (IDCC) model to manage the multicenter clinical research setting and named it the plasma coordination center. Here we describe the plasma coordination center’s rapid CCP qualification, acquisition, and distribution of control plasma and CCP in the linked Post Exposure Prophylaxis and Early Treatment outpatient clinical trials, using the IDCC model described above and an FDA-compliant research randomization and tracking database in the outpatient setting, during the COVID-19 pandemic. These two randomized clinical trials sought to answer the questions of whether CCP could prevent infection in exposed individuals (Post Exposure Prophylaxis) [11] or reduce severe disease in symptomatic, infected individuals (Early Treatment) [12] and have been previously described.

## Methods

### Study plasma sourcing and qualification

The investigational product, CCP, was convalescent plasma containing antibodies to SARS-CoV-2 obtained from individuals who had recovered from COVID-19 [12]. The CCP was sourced by donor screening programs at Johns Hopkins University, Endeavor Health (formerly Northshore University HealthSystem), Luminis Health Anne Arundel Medical Center, New York Blood Center Enterprises, and ImpactLife blood center for nationwide distribution; four other enrolling sites – University of Utah, University of California Los Angeles, University of Cincinnati, and Nuvance Health (New York and Connecticut) – provided CCP for local, single-site use.

Under an IND19727 study protocol, the presence of SARS-CoV-2 antibodies was confirmed in eligible donors after a 1:320 plasma dilution was positive on one of three validated spike-protein enzyme-linked immunosorbent assays (ELISAs), including the Anti-SARS-CoV-2 ELISA (IgG) (Euroimmun), the Vitros COVID-19 IgG Assay (Ortho Clinical Diagnostics), and the COVID-19 ELISA IgG Antibody Test (Mount Sinai Laboratory), in a Clinical Laboratory Improvement Amendments (CLIA) certified laboratory. Potential donors who met these qualification standards were referred to an FDA-registered blood center where donors were evaluated according to current blood donation requirements; plasma was then collected and licensed as fresh frozen plasma (FFP), frozen within 8 hours, or plasma frozen within 24 hours of phlebotomy (PF24) [13,14]. After both qualification and transfusion, donor CCP antibody levels were characterized in research laboratories by determining their titers with full-length ancestral spike and live virus growth neutralization assays and Euroimmun arbitrary unit reactivity at manufacturer’s recommended dilution of 1:101 [15,16]. Control plasma was given to participating site hospital blood banks from FDA-registered blood centers as FFP or PF24 collected before January 1, 2020, or confirmed as SARS-CoV-2 seronegative, if collected later.

### Plasma coordination center organization

A robust plasma coordination center team was established to act as the resource center for site blood bank directors and personnel, plasma suppliers, specimen repository personnel, and the trial leadership (Table 1). The Johns Hopkins Trial Innovation Center (TIC) served as the clinical operations center supporting the plasma coordination center. The principal investigators were the regulatory sponsors and negotiated plasma acquisitions. The pharmacist/project manager implemented operational systems to streamline shipments and oversee inventory distribution and monitoring and analysis programs to track inventory and protocol compliance. Other team members vital to trial safety and success included unblinded monitors and a coordinator for support activities. The center team was available via email or telephone and through an interactive web application. Transfusion and blood bank co-investigators liaised with leadership and advised the coordination center team.

**Table 1.** Roles and activities of a plasma coordination center and blood banks supporting two COVID-19 outpatient convalescent plasma (CCP) trials at 26 centers

Role	Responsibilities
Plasma control center charge	- To ensure regulatory compliance and establish standardized procedures for the procurement, storage, handling, and dispensing of plasma products - To implement safety and control measures to maintain plasma quality and keep meticulous records to ensure traceability and accountability of the investigational product throughout the trial process
Principal Investigator	FDA IND sponsor activities Plasma donor procurement IRB submissions and changes in research Financial resources management
Director, Clinical Operations	Plasma center integration with other teams LOCATOR* database construction Trial MOP/SOP^†^ materials production Training module production
Pharmacist/Project Manager	Site blood bank communications and interactions Donor and courier vendor supply chain activities ABO group inventory allocations QA monitor oversight LOCATOR database analyses Startup and close-out activities
Coordinator	LOCATOR database maintenance and report generation Randomization activities General support
Unblinded Monitor	Regulatory documentation & compliance activities LOCATOR data reviews LOCATOR data cross audits with EDC data
Blood Bank Liaison	Blood bank regulatory MOP/SOP production Blood bank regulatory monitoring Randomization, education, and training advisement
Qualified Repository Manager	CCP antibody testing and registration CCP specimen receiving and shipping activities
Site Blood Bank Director	Site-specific education and training Site-specific inventory and LOCATOR database oversight Site-specific IRB and financial management
Site Blood Bank Personnel	LOCATOR database inventory and dispensing activities IP dispensing for transfusion Supply chain maintenance activities

* MOP/SOP = Manual of Procedures/Standard Operating Procedures.

^†^LOCATOR = Investigational Product Management Standard Operating Procedure.

A secure web application, LOCATOR (Investigational Product Management Standard Operating Procedure), was developed by the TIC clinical operations center team within the Prelude electronic data capture system (Prelude LLC, Austin, TX) to create simple online data collection forms, facilitate online tracking of plasma sourcing, qualification, and distribution, and to manage randomization and transfusion operations. The servers were housed in two TIER III, SSAE-18 certified data centers in Austin, Texas, and protected by multiple firewalls and a LINUX Operating System that ensured high-level security. Data were encrypted during transit, and SSL (Secure Sockets Layer) was used for all public-facing internet provider addresses. Server data were stored on redundant RAID drives, with study data backed up hourly to an off-site repository. An annotated blank CRF and codebook (online SRD Field Spec) were maintained on file throughout the trials.

The system included built-in features for electronic data collection, electronic signatures, and a time-stamped audit trail that enabled ongoing reconstruction of the course of events relating to the creation, modification, and deletion of each plasma record. All features were in adherence with the FDA Guidance for Industry for Computerized Systems Used in Clinical Trials (May 2007) and the Electronic Records/Electronic Signatures rule (21 CFR Part 11, Electronic Records). Additionally, the center followed the FDA guidance on Good Clinical Practice (ICH E6(R2), Mar 2018) to ensure the efficient collection of quality data.

The system was fully validated and compliant with General Data Protection Regulation, Annex 11, and Health Insurance Portability and Accountability Act regulations. The LOCATOR system development and release was managed by Prelude Dynamics, LLC, a life science software company supporting electronic clinical data management platforms since 2003. Prelude systems have two processes for development and testing, one for the core software architecture and one specific to the studies. The basic data collection system was validated independently, outside the context of the study. The study testing was done with the data managers to verify that the underlying functionality was working as required for the study. Prelude Quality Assurance (QA) SOPs controlled the performance of validations and testing. For these studies, the QA department performed a series of checks before data managers were given access.

During user acceptance testing (UAT), the data managers tested all fields of entry in a training version of the study. The UAT team tested all fields, constraints, and calculations, including question labels, data types, valid ranges, mandatory fields, branching logics, and cross-form checks. The system supported an integrated feedback function where the data managers entered feedback directly into the training version. During the data manager reviews, the feedback function was used to request changes to LOCATOR and each change was tracked and archived through to verification and acceptance. The data managers verified that all outstanding change requests were addressed and entered additional feedback when necessary. The data managers then signed off on LOCATOR’s build, authorizing live transition to the Live study. All UAT activities are time-stamped and preserved in a permanent archive.

The roles and responsibilities of the plasma coordination center staff and site personnel involved in IP management were organized and documented in LOCATOR to support online operations and remote QA monitoring (see Table 1). Blinded trial monitors external to the plasma coordination center were responsible for monitoring all usual aspects of the trials, except IP management where specially assigned unblinded monitors were responsible for monitoring the IP inventory and usage. Unblinded site blood bank staff documented IP orders, shipment, receipt, inventory level, storage conditions, over-labeling, transfer, return, and destruction directly in the LOCATOR database with secure password access.

Although site blood bank personnel are well-skilled at blood product current Good Manufacturing Practice standards and local and federal regulatory agency standards, as this was an Investigational New Drug (IND)-approved trial, we presented study-specific training as Good Clinical Practice (GCP) to ensure the reliability and robustness of data generated by the study. Site blood bank personnel and the site clinical teams were trained in IP management procedures to ensure compliance with GCP guidelines. Training also covered chain-of-custody events, including IP re-ordering, receipt, storage, dispensing, over-labeling, return, and discard, as well as maintaining blinding and the LOCATOR web application.

Trial planning and site startup occurred in parallel. While sites were being selected, project managers worked with the sponsoring-site blood bank liaison and trial leadership blood bank directors to manage IND matters in compliance with ICH-GCP E6 guidelines and write the investigational manuals of operation and training modules, and the blood bank investigators and clinical operations designed the LOCATOR platform for the plasma coordination center. While sites were in their startup and training phase, the pharmacist/project manager and coordinator were assigned full-time to the center to liaise with the single institutional review board, build database reports, and train monitors. Through a clinical trial employment agency (Actalent, Hanover, MD), multiple unblinded QA monitors were hired to monitor both plasma coordination center activities and the data being entered by enrolling site blood bank personnel.

### Distribution of study plasma

We used a decentralized distribution model. Plasma was not kept at the plasma coordination center or stored at a single blood establishment. Based on the distribution frequency of blood group A antigen, blood group B antigen, and blood group O lacking A and B antigens, abbreviated as ABO, a minimum supply was sent to each site upon study activation and then replenished as depleted. The trial purchased substantial amounts of qualified plasma from donor/blood bank centers who agreed to keep the trial-owned units safely in quarantine and ship overnight to where the units were needed to replenish minimum site inventories. Daily, the plasma coordination team communicated with site blood bank directors, plasma donor centers, and courier services to ensure inventory levels and the safe delivery of temperature-sensitive plasma. Sites placed orders directly with the pharmacist/project manager who confirmed the site inventory in LOCATOR, then coordinated a temperature-controlled courier shipment from a donor center to the site blood bank. All steps of the distribution were recorded in LOCATOR.

### Randomization, blinded labeling, and transfusion

Trial plasma was sequestered at each site according to local blood bank policies. The LOCATOR system generated an online randomization that sent an automated email to the site’s blood bank. Unblinded blood bank personnel were able to select the correct CCP or control product and cross verify the patient’s ABO group with the plasma type [17] from the sequestered trial supply. Participants could receive plasma of the same ABO group or an ABO-compatible plasma type, except for pregnant participants for whom identical matching was required. The plasma product was thawed, over-labeled in a manner to blind its identity, and then provided to the infusion team in standard plasma unit bags with an FDA-format ISBT label specifying its IND status and storage requirements [18,19]. Control plasma and CCP were identical in appearance except for different anticoagulants used during the collection process printed on the over-label. The allocated plasma was transfused by the site study team according to health system standard operating procedures.

All plasma units received were allocated for both the Infection Prevention (CSSC-001) and Early Treatment (CSSC-004) trials from June 2020, with joint inventory maintenance, until March 2021. After completion of the CSSC-001 trial in March 2021, the remaining units were transferred for use as CSSC-004 trial inventory.

Datasets from LOCATOR were exported every two days to create multiple reports, including inventory counts, ABO group allotments, shelf-life expectancy, and upcoming expirations, using Python (Python Software Foundation, Beaverton, OR) and SAS (SAS Institute Inc., Cary, NC) programing tools. This ensured that IP was handled with care and that records were maintained within the database in accordance with federal, state, and local regulations. These semiweekly exports were reported at weekly meetings attended by the leadership and program officers from each funding agency.

Data sets were exported from LOCATOR to Microsoft® Excel to generate Sankey diagrams (http://sankeymatic.com/build/). The Sankey tool produced a visual chart of convalescent and control plasma journeys from donation to transfusion, permitting source and destination traffic comparisons. For comparisons of antibody levels, *P*-values less than 0.05 were considered statistically significant. Analyses were performed using GraphPad Prism 10 (GraphPad Software).

## Results

Overall, the plasma coordination center utilized 3,159 donor units to support 1,351 trial transfusions throughout the U.S. in 16 months for the 170-participant Infection Prevention randomized controlled trial (RCT) [10] and the 1,181-participant Early Treatment [11] RCT.

### Pandemic study site activations

**Figure 1.**
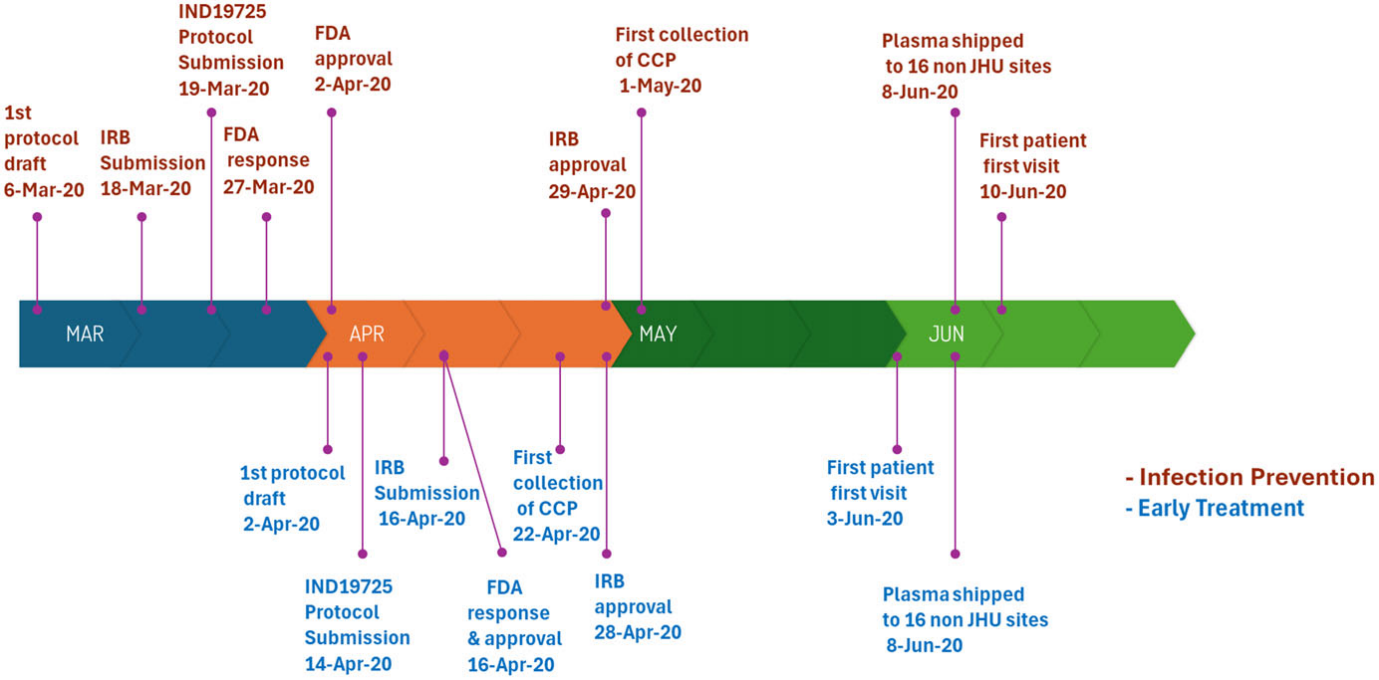
Pandemic research study startup. The events for the postexposure prophylaxis study for infection prevention are shown above the 4-month timeline for first patient visit and nationwide site activation with donor plasma distribution. Similar events for the outpatient early treatment study are shown below the timeline.

The outpatient postexposure prophylaxis Infection Prevention trial was conceived in early March 2020, where within two weeks the protocol was submitted to each local IRB and the FDA under the IND application (Figure 1). Our FDA IND19725 was granted on April 2, within two weeks of submission and less than one month after study conception. Institutional review board approval followed in a month, and the first patient visit was on June 10, 2020. The outpatient Early Treatment protocol followed a likewise pathway under the same IND19725, with protocol conception, FDA, and IRB approvals within a month and first patient visit on June 3, 2020. Donor CCP for study participants was initially collected in late April and early May 2020 and continued until March 2021. After the initial participant transfusions at the sponsoring center the first week of June 2020, site blood banks nationwide received study-qualified control plasma and CCP as a final activation benchmark.

### CCP sourcing, qualification, distribution, and transfusion

We obtained 3,159 plasma units from 29 donor collection centers, comprising 1,459 (46%) convalescent and 1,700 (54%) control plasma units. CCP was sourced from Maryland, Connecticut, Iowa, Illinois, Ohio, California, New York, New Jersey, Delaware, and Utah. Control plasma was principally sourced from American Red Cross, NYBCE (New York Blood Center Enterprises), military blood banks in Washington DC and the states of Hawaii, Illinois, Pennsylvania, New Jersey, New York, Delaware Texas, California, Ohio, and Connecticut. Control plasma was collected either in 2019 or was SARS-CoV-2 spike antibody negative.

In April 2020, the FDA approved the IND19725 protocols which specified CLIA positive SARS-CoV-2 antibodies by ELISA after a 1:320 dilution. After July 2021, the new March 9, 2021, FDA Emergency Use Authorization (EUA) threshold (Euroimmun ratio > 3.5) for high-titer hospital plasma was used for remaining CCP transfusions. More than 80% of the research-qualified plasma units also met the March 9, 2021, EUA high-titer qualifications of the FDA. Across both studies, some apheresis donations went to multiple recipients: 403 individual donors supplied 679 units transfused to recipients.

**Figure 2.**
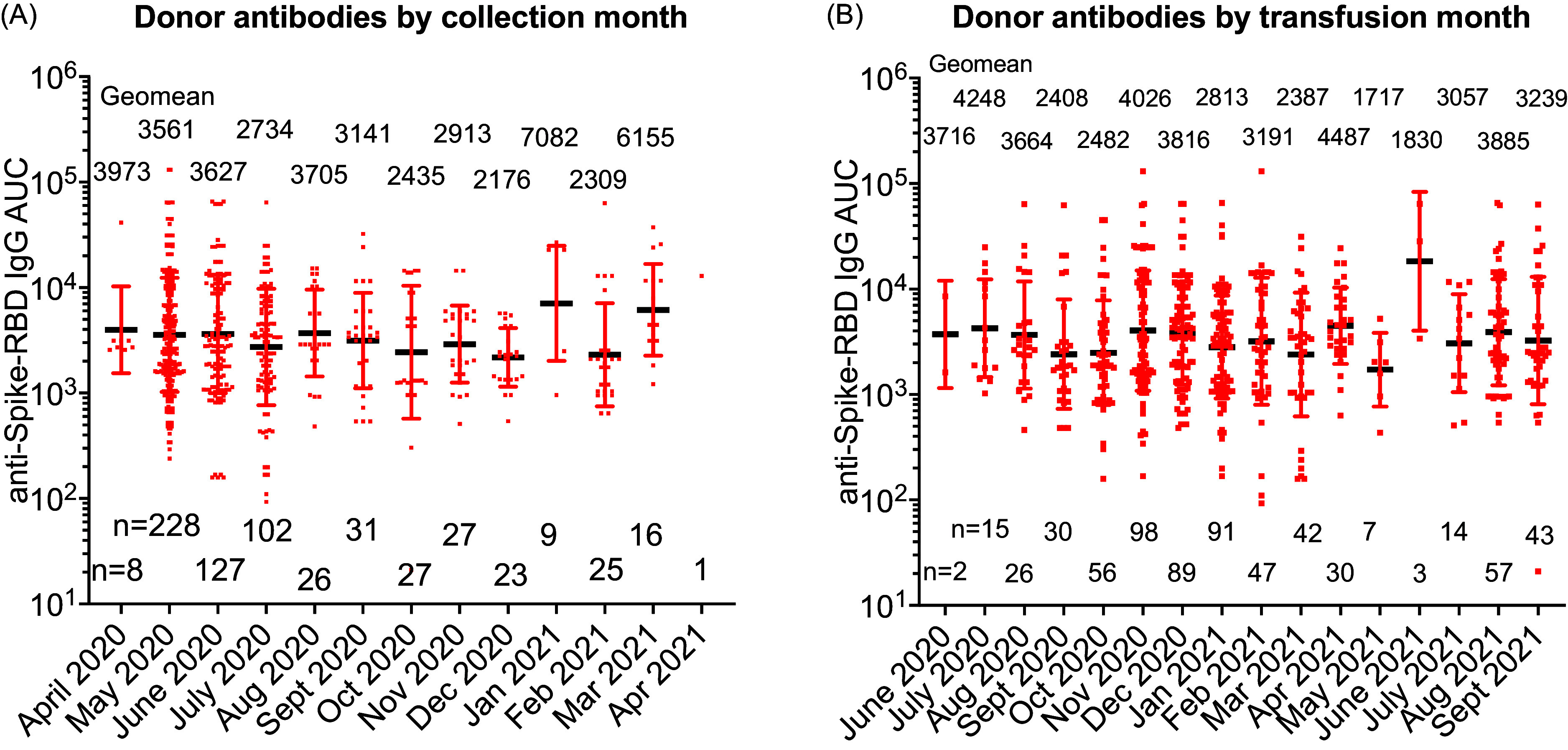
Viral specific antibody levels by collection date and transfusion date. Anti-spike receptor binding domain immunoglobulin G area under the curve (Anti-Spike-RBD IgG AUC) levels among 650 donor units transfused into recipients segregated by A) month of collection and B) study month of transfusion. The transfusion number by month and anti-Spike-RBD IgG AUC geomean are depicted. Comparison of log10 transformed values indicated no differences by 2-way ANOVA with multiple comparisons.

**Figure 3.**
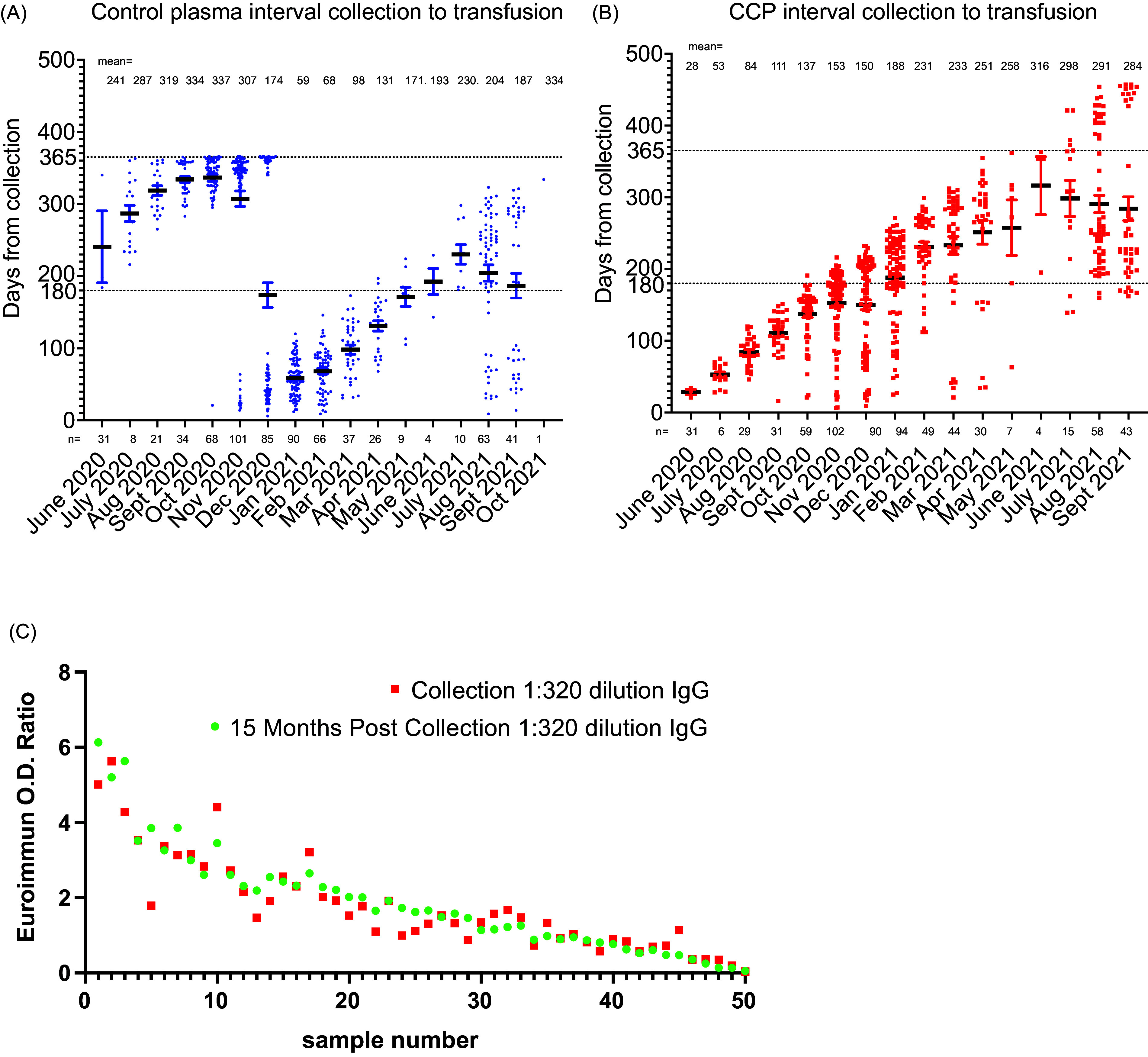
Time from collection to transfusion for COVID-19 convalescent plasma (CCP) or control. The 2019 control plasma was used first, then the 2020 collected SARS-CoV-2 seronegative control plasma. The interval from donor collection date to recipient transfusion date for (A) control plasma and (B) CCP. (C) FDA granted CCP a 3-month shelf-life extension to 15 months based upon antibody stability data submitted under IND19725. Donor plasma samples collected in early 2020 were diluted 1:320 for the Euroimmun assay. Fifteen months later, the same samples were thawed with repeat SARS-CoV-2 S1 Spike antibody levels measured after 1:320 dilution.

After study completion, a retrospective analysis of the viral-specific antibody levels over the 10 collection months as well as during the 16 transfusion months showed that antibody levels remained constant (Figure 2). The interval from collection to transfusion date was less than 365 days for control plasma (Figure 3A). The time from collection to transfusion in the CCP group was 177 days for 17 patients who were hospitalized after transfusion and 195 days for those not hospitalized (Figure 3B). We anticipated a shortfall in qualified CCP units towards the end of the Early Treatment study and performed a 15-month stability study on fifty CPP units stored at –80°C, which demonstrated no change in antibody levels. Based on these data, the FDA granted an extension from 12 months to 15 months for the IND19725 designated CCP to allow units past the standard frozen plasma shelf life of 12 months to be transfused for another three months (Figure 3C).

**Figure 4.**
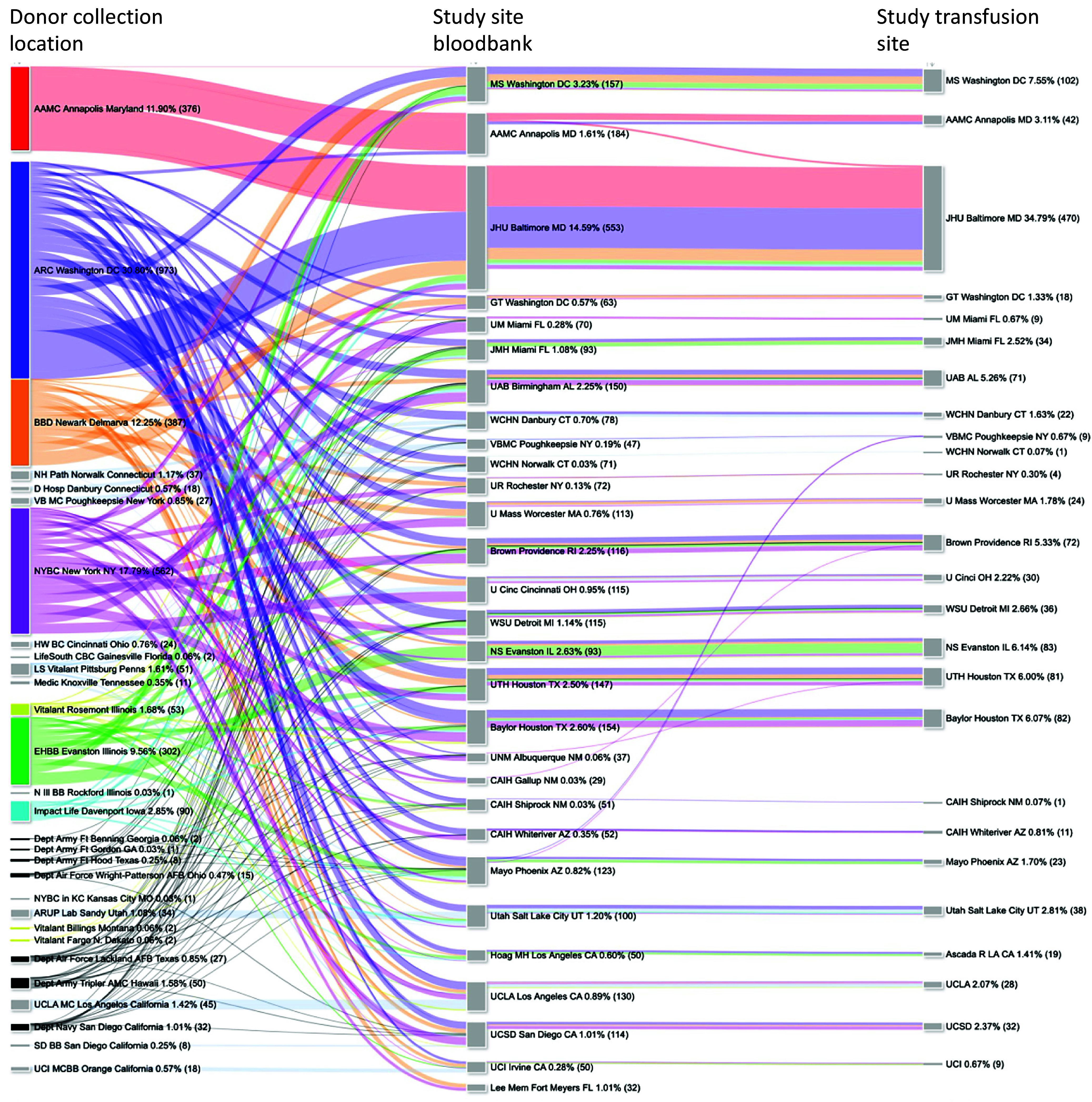
Flow of both COVID-19 convalescent plasma (CCP) and control plasma units from donor collection centers to site blood banks and 26 transfusion locations. The width of each line in the figure indicates the volume or the quantity of plasma units distributed, while the line color corresponded to the U.S. census collection region by blood unit WDIN. After name of blood banks or study site is the column percent of total (absolute number). EHBB = Endeavor Health Blood Bank, NS = Northshore, NYBC = New York Blood Center Enterprises, Hoag MH = Hoag Memorial Hospital in Newport Beach, CAIH = Center American Indian Health, MS Washington DC-Medstar, WSU = Wayne State University Detroit, WCHN = Western Connecticut Health Network aka Nuvance, AAMC = Anne Arundel Medical Center, GT = Georgetown.

Each site blood bank maintained an inventory of between 12 and 20 units with an ABO blood group ratio target of 1 AB, 1 B, 2 A, and 2 O for each arm. The first Sankey chart plots plasma sourcing and distribution to individual sites during the entire trial (Figure 4). A total of 3,159 plasma units were collected, qualified, and distributed to 29 blood banks for 1,351 transfusions, all facilitated via the LOCATOR platform. Anne Arundel Medical Center in Maryland, New York Blood Center Enterprises, and Endeavor Health in Evanston, Illinois supplied both control and CCP. ImpactLife in Iowa supplemented CCP in the summer of 2021. The American Red Cross in Washington, DC supplied control plasma, which was distributed to all study sites. The Department of Defense also supplied control plasma. Small numbers of IP and control plasma units were redistributed between sites near the end of the study to keep up with demand.

**Figure 5.**
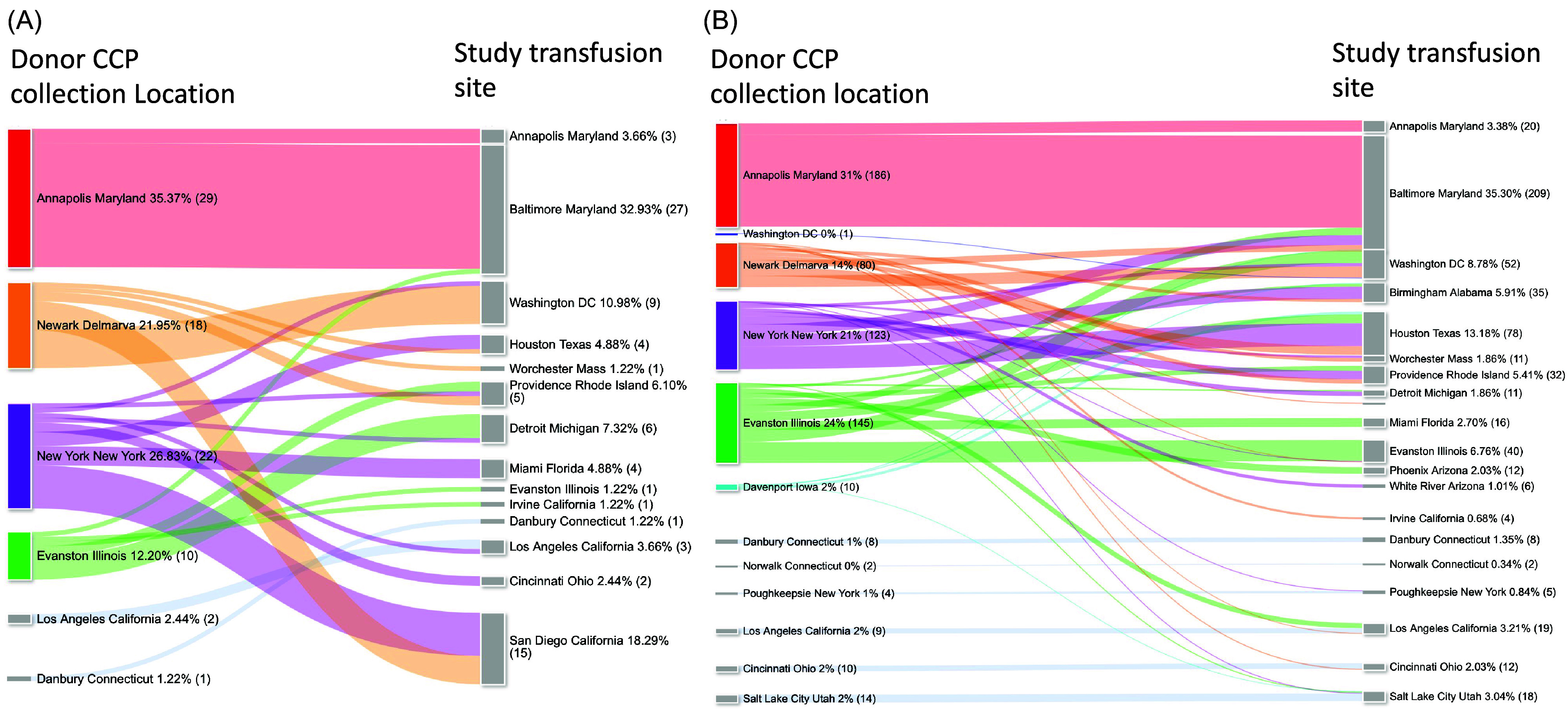
Flow of COVID-19 convalescent plasma (CCP) plasma units per study. (A) Infection Prevention RCT with 70 unique CCP donations were acquired and transfused to 82 recipients. CCP was collected at regional supply centers: Annapolis, Maryland (35%), New York Blood Center, NY, NY (27%), Blood Bank of Delmarva, Newark, NJ (22%) and Evanston, Illinois (12%). Additionally, locally sourced plasma was collected in Los Angeles, California, Danbury and Norwalk, Connecticut, and Poughkeepsie, New York (4%). (B) Early treatment RCT where 333 unique CCP donations were acquired and transfused to 592 recipients. CCP was collected at regional supply centers: Annapolis, Maryland (31%), Evanston, Illinois (24%), the New York Blood Center, NY, NY (21%), and Blood Bank of Delmarva, Newark, NJ (14%), ImpactLife, Davenport, Iowa (2%). The remaining 8% (46) units were locally sourced at study sites. After name of blood banks or study site is the column percent of total (absolute number).

Having CCP distributed from both nationwide and local donations, we used Whole Blood - Donation Identification Number codes to identify and match donor collection locales to recipient transfusion sites. In the Early Treatment trial, there were 366 of 592 (62%) regional matches (Figure 5). Of those hospitalized, 15 of 17 (88%) were matches of donor and recipient from the same region; only two hospitalizations in the CCP group received a transfusion from outside the local community.

## Discussion

Working with experimental blood products is hard for many blood establishments and there is a scarcity of experience in most blood establishments using plasma as IP [3]. However, the complexities become more manageable when you mobilize a core team with knowledge of the regulatory requirements that govern clinical research and basic pharmacy processes for using drugs in randomized clinical trials. The traditional central research pharmacy team can be mirrored in the configuration of a central plasma coordination team charged with organizing blood product procurement from donor centers and distributing it to site blood bank personnel who will take on the eventual dispensing of the product to trial investigators for transfusion into enrolled participants. Likewise, the integration of a blood product across multiple enrolling sites requires the usual planning and scheduling of activities, transactions, operations, and organization of site investigational pharmacies, the difference being that the IP will be managed in hospital blood banks rather than the usual local research pharmacies.

Our experience shows that it is possible to rapidly unite clinical trial research personnel in academic centers with blood bank personnel across the U.S. to create a temporary network capable of qualifying a potentially life-saving blood product treatment in a chaotic pandemic environment. Once the protocol was created, it was clear that the project needed a plasma coordination center that would be responsible for the testing of high levels of antibodies in CCP, the procurement and maintenance of sufficient treatment and control plasma units, and the distribution and monitoring of the plasma inventory.

Once the plasma coordination center was created and staffed with a full-time pharmacist and monitoring team performing their crucial roles of directing plasma transfers from donation storage centers directly to site blood banks, a second decision was made to have site IP inventory remain physically under the management of each blood bank director rather than a site pharmacy. Therefore, we worked with blood bank teams to flexibly adapt inventory decisions based on the specific dynamics of each participating blood bank. Protocol and blood bank regulation compliance issues were addressed by trial leadership, the site principal investigators, and the blood bank directors working together.

Effective communication played a crucial role in the smooth implementation of the trial and management of challenges. The plasma coordination center team, including unblinded monitors, maintained daily communication with site blood banks through the interactive LOCATOR platform, as well as via emails and phone calls, to oversee the chain of custody of the IP and its regulatory documentation.

Balancing site blood bank regulations and the FDA, GCP/ICH guidelines for pharmacies, and clinical trials added additional layers of complexity. Oversight of the plasma IP required identification of adequate amounts of ABO-typed control plasma and CCP at the 29 donor/blood bank locations. In addition, managing inventory of specific ABO groups and expiration dates required flexibility to be granted to the 26 site blood banks. Shipping of research study IP, under strict blood bank regulations, was indeed innovative. The creation of a procurement core and distribution of specific blood products through an investigational “pharmacy-like” model allowed us to maintain compliance with blood bank regulations while ensuring appropriate products were available for research study distribution.

Daily assessments and weekly reporting of minimum inventory requirements at all sites and centers matched to COVID-19 surges were priorities for the plasma coordination center team. ABO blood group supply uncertainty played a significant role in inventory management, and quick decisions about product redistribution between sites were necessary. In the pandemic situation, we were unable to forecast study enrollment patterns or predict product use based on historical data on the amount of plasma used at each site. Maintaining adequate levels of IP and control plasma at the source distribution centers and among the enrolling centers was accomplished by banking excess study-qualified plasma at the source distribution centers and reviewing distribution levels every week with the entire trial leadership. LOCATOR provided the trends and acquisition requests, and algorithms were developed to determine weekly minimum threshold inventories.

In the fall of 2020, in hospitalized patients with death as the endpoint, a study reported that median titer CCP demonstrated better outcomes with regionally matched plasma [20]. In contrast, for these outpatient studies with CCP characterized with high antibody levels transfused early from onset of illness, regional mismatches implicating variant mismatch did not influence the hospital primary endpoint, with the caveat that only 17 hospitalizations after CCP transfusion is a small number.

## Conclusion

The trial benefited from altruistic individuals who wanted to contribute to a COVID-19 solution by donating convalescent plasma. This turned into a national endeavor, the National Convalescent Plasma Project (CCPP19.org), that was guided by trial leaders. We were able to unite with blood banks across the U.S. and assemble a temporary plasma coordination center to rapidly test a life-saving treatment in a rapidly changing COVID-19 pandemic environment.

Using previous trial experience, we modeled our plasma coordination center on IDS best practices [16], creating the IDCC model, and built a Part 11 compliant data collection and inventory tracking web system (LOCATOR, Prelude Dynamics, Austin, TX) mimicking commercial pharmacy clinical trial software and adding blood bank regulation features. Functioning as a unique intersection of investigative pharmacy and blood bank inventory trackers, the LOCATOR system provided a central inventory databank accessible to the plasma coordination center and the blood bank personnel at each enrolling center. The single-source inventory platform allowed real-time monitoring of inventory and distribution from collection centers to hospital blood banks to facilitate patient treatment.

As the world is currently amid an avian influenza outbreak that is already involving several mammalian species and threatens humanity, our experience, as detailed here, could provide a roadmap for the establishment of similar coordination centers that collaborate with local blood banks to support the clinical testing, inventory management, and distribution of convalescent plasma. The model could serve as a template for subsequent public health emergencies.
